# 单倍型造血干细胞移植治疗侵袭性NK细胞白血病五例临床观察

**DOI:** 10.3760/cma.j.issn.0253-2727.2021.07.010

**Published:** 2021-07

**Authors:** 晶 夏, 苏宁 陈, 正明 金, 晓文 唐, 峰 陈, 骁 马, 瞄 苗, 德沛 吴

**Affiliations:** 苏州大学附属第一医院，江苏省血液研究所，国家血液系统疾病临床医学研究中心，苏州 215006 National Clinical Research Center for Hematologic Diseases, Jiangsu Institute of Hematology, The First Affiliated Hospital of Soochow University, Suzhou 215006, China

侵袭性NK细胞白血病（ANKL）是一种与EB病毒（EBV）感染密切相关的NK细胞淋巴增殖性疾病，以系统性NK细胞浸润和侵袭性临床过程为特征。ANKL好发于中青年，男性稍多于女性，在亚洲和拉丁美洲多见[1]–[2]。临床上具有起病急、进展快、预后差等特点。EBV感染几乎存在于所有ANKL患者中，且被认为是ANKL的重要致病原因之一[3]。ANKL对多种化疗方案反应不佳，短期内出现多脏器功能衰竭，中位生存期约为2个月[4]。我中心自2015年1月至2018年11月应用单倍型造血干细胞移植（haplo-HSCT）治疗5例ANKL患者，获得了较好疗效。

## 病例与方法

1. 病例：以2015年1月至2018年11月在我科行haplo-HSCT的5例ANKL患者为研究对象。所有患者均经骨髓形态学及病理学检查，同时行免疫组织化学染色、流式细胞术、染色体核型等检测确诊为ANKL，诊断标准符合2016年造血与淋巴组织肿瘤WHO分类对ANKL的形态学描述、免疫标记特点和分子生物学指标。所有5例患者均无HLA全相合供者。

2. 移植前治疗：4例患者采用甲氨蝶呤（MTX）+异环磷酰胺+培门冬酶+依托泊苷+糖皮质激素（改良SMILE方案）进行诱导治疗，其中1例加用芦可替尼控制细胞因子风暴。1例患者采用培门冬酶+吉西他滨+奥沙利铂（P-Gemox方案）诱导化疗。5例患者选用原方案巩固化疗1～5个疗程。2例患者移植前处于第1次完全缓解（CR_1_）状态，2例患者处于部分缓解（PR）状态，1例未缓解（NR）患者以hallo-HSCT作为挽救治疗。

3. 移植方案：4例患者采用改良全身照射（TBI）+环磷酰胺+兔抗人胸腺细胞免疫球蛋白方案：司莫司汀250 mg·m^−2^·d^−1^+羟基脲40 mg/kg每12 h 1次，−10 d；TBI总量12 Gy，−9～−7 d分次照射；阿糖胞苷2 g·m^−2^·d^−1^，−9 d、−8 d；环磷酰胺1.8 g·m^−2^·d^−1^，−4 d、−3 d；兔抗人胸腺细胞免疫球蛋白2.5 mg·kg^−1^·d^−1^，−5 d～−2 d。1例患者采用改良白消安/环磷酰胺（Bu/Cy）+兔抗人胸腺细胞免疫球蛋白预处理方案，以白消安（0.8 mg/kg每6 h 1次静脉滴注，−7～−5 d）取代TBI，其余不变。

采用环孢素A（CsA）+短程MTX+霉酚酸酯（MMF）预防GVHD。CsA 3 mg·kg^−1^·d^−1^，−9 d开始静脉滴注，肠道功能恢复正常后改为口服，维持血药浓度200～300 µg/L。MTX 15 mg/m^2^，+1 d；10 mg·m^−2^ ·d^−1^，+3 d、+6 d、+11 d。MMF 30 mg·kg^−1^·d^−1^，−9 d开始口服，+30 d开始减量，移植后2～3个月停用。应用肝素、前列腺素E_1_预防肝静脉闭塞病（HVOD），常规预防细菌、真菌、巨细胞病毒及卡氏肺孢菌感染。

4. 随访：随访截至2020年10月1日，移植后中位随访23（2～69）个月，无失访病例。每2周检测血常规、每1～3个月检测骨髓象以及微小残留病（MRD）。总生存（OS）时间为造血干细胞回输到随访截止或患者死亡的时间。

## 结果

1. 一般资料及临床表现：5例ANKL患者中男3例，女2例，中位年龄36（28～44）岁。发热（4例）为ANKL患者最常见的就诊原因，脾大（5例）是最常见的体征。初诊中位白细胞计数为3.8（2.9～33.4）×10^9^/L，发病时多数表现为轻中度贫血，血小板计数为（6～78）×10^9^/L。所有患者的乳酸脱氢酶均升高且存在肝功能异常。所有患者血清EBV-DNA均为阳性。详见表1。

**表1:** 5例侵袭性NK细胞白血病（ANKL）患者诊断时一般资料

例号	性别	年龄（岁）	HGB（g/L）	WBC（×10^9^/L）	PLT（×10^9^/L）	骨髓原始细胞比例（％）	发热	脾大	EBV	乳酸脱氢酶（U/L）	肝功能异常
1	男	44	95	3.8	6	84.0	是	是	阳性	2247	是
2	男	34	101	9.5	78	19.5	是	是	阳性	540	是
3	男	37	147	33.4	67	38.5	否	是	阳性	1796	是
4	女	36	119	2.9	64	41.0	是	是	阳性	550	是
5	女	28	69	2.4	36	20.5	是	是	阳性	2095	是

2. 预处理相关不良反应及并发症：5例患者在预处理期间和之后发生1～3级呕吐伴腹泻，对症处理后症状消失。粒细胞植入前3例患者发生感染（血流感染、肺部感染、肠道感染各1例）。2例患者在血小板植入前偶有皮肤黏膜出血点。2例患者发生一过性癫痫，经对症治疗后痊愈。未发生HVOD。移植后2例患者发生CMV血症，经积极治疗后病毒拷贝数转阴，2例移植后复发时EBV-DNA再次转阳。

3. 造血重建：输注供者单个核细胞（MNC）中位数为15.20（11.92～20.21）×10^8^/kg，CD34^+^细胞中位数为5.31（3.97～6.11）×10^6^/kg。所有患者均获得造血重建，中位粒细胞植入时间为移植后12（11～14）d，中位血小板植入时间为移植后13（12～25）d。所有患者在移植后30 d经STR、性染色体荧光原位杂交及血型检测证实获得完全供者植入。详见表2。

**表2:** 5例侵袭性NK细胞白血病（ANKL）患者造血干细胞移植情况及转归

例号	移植前状态	预处理方案	GVHD预防	回输单个核细胞（×10^8^/kg）	回输CD34^+^细胞（×10^6^/kg）	粒细胞植活（d）	血小板植活（d）	急性GVHD	慢性GVHD	病毒感染	转归	移植后存活时间（月）
1	CR_1_	TBI/Cy	CsA+MMF+MTX	11.92	5.32	14	25	Ⅱ度肝脏	广泛型	CMV-DNA阳性	存活	23
2	PR	TBI/Cy	CsA+MMF+MTX	12.82	4.24	11	14	无	无	EBV-DNA阳性	死亡	13
3	NR	Bu/Cy	CsA+MMF+MTX	17.80	6.11	11	12	无	局限型	CMV-DNA阳性	存活	69
4	PR	TBI/Cy	CsA+MMF+MTX	20.21	5.31	12	13	无	无	EBV-DNA阳性	死亡	2
5	CR_1_	TBI/Cy	CSA+MMF+MTX	15.20	3.97	12	14	Ⅱ度皮肤	无	无	存活	28

注：CR_1_：第1次完全缓解；PR：部分缓解；NR：未缓解；CsA：环孢素A；MTX：甲氨蝶呤；MMF：霉酚酸酯；GVHD：移植物抗宿主病；TBI：全身照射；Cy：环磷酰胺；Bu：白消安；CMV：巨细胞病毒；EBV：EB病毒

4. GVHD发生情况：2例患者发生Ⅱ度急性GVHD（皮肤、肝脏各1例），经甲泼尼龙、普乐可复、MTX及抗CD25单抗治疗后好转。2例患者发生慢性GVHD（局限型、广泛型各1例），给予甲泼尼龙、芦可替尼等治疗后好转（表2）。

5. 移植疗效：随访至2020年10月1日，中位随访23（2～69）个月，5例患者中3例存活，2例死于疾病复发。

## 讨论

ANKL最早由Imamura等于1990年发现，是一种与EBV感染密切相关的高度侵袭性的疾病。ANKL归为成熟的NK/T细胞肿瘤，见于亚洲和拉丁美洲，多在中青年发病，中位发病年龄30岁，男女发病率无差异。突出特点为外周血或骨髓中NK细胞来源的大颗粒淋巴细胞增生，进展迅速，病情凶险，短期内出现多脏器功能衰竭、噬血细胞综合征，对化疗反应不佳，中位生存期小于2个月[5]–[6]。本组患者以持续发热伴肝脾肿大、肝功能异常、血清EBV-DNA阳性为主要特征，病程进展迅速。

因为发病率低、大多数患者病情进展快速，ANKL目前仍缺乏标准治疗方案。CHOP或CHOP样传统化疗方案对少数患者有效，但短期内易复发[4]。这一化疗耐药性目前认为是由P-glycoprotein（—种由MDR1基因编码的在NK细胞中表达的跨膜外输泵）所介导[7]–[8]。而左旋天冬酰胺酶（L-asp）不受糖蛋白P的影响，从而对NK/T细胞淋巴瘤有效。有多个研究证实含L-asp的联合化疗方案可明显提高疾病的缓解率[9]–[11]。培门冬酶作为聚乙二醇包裹的天冬酰胺酶，不仅具有与L-asp相同的抗肿瘤作用，还有半衰期长、用药方便、不良反应少等优点。本组病例均采用含培门冬酶的方案，2例患者达到CR，2例达到PR。

ANKL患者病程中常出现噬血细胞综合征（HPS），属于获得性HPS。Ishida等[12]报道34例ANKL患者，其中19例（56％）病程中发生HPS。免疫系统的异常激活及炎性细胞因子的大量分泌在HPS发病机制中具有关键作用。HPS有关细胞因子大都通过激活JAK/STAT信号通路而活化。在小鼠HPS模型中，国外部分研究证实Janus激酶抑制剂芦可替尼抑制信号传感器和激活转录单基因表达，限制CD8^+^T细胞的活化，从而减少促炎细胞因子的产生以改善病情[13]–[14]。早期使用芦可替尼可缩短细胞因子风暴持续时间，其安全性良好且可能使患者长期生存获益[15]–[16]。例1在病程中出现HPS，我们采用芦可替尼联合SMILE方案，病情得到快速改善。

鉴于ANKL高度恶性、易复发的特点，我们对本组5例ANKL患者给予haplo-HSCT治疗。中位随访23（2～69）个月，3例存活，2例死于复发，未发生移植相关死亡，提示haplo-HSCT针对这类患者是有效、安全的，但还需要大样本、多中心数据进一步证实。
